# Vigorous intermittent lifestyle physical activity (VILPA) and mortality risk among US adults: a wearables-based national cohort study

**DOI:** 10.1186/s12966-026-01876-2

**Published:** 2026-01-30

**Authors:** Nicholas A. Koemel, Matthew N. Ahmadi, Raaj Kishore Biswas, Cecilie Thøgersen-Ntoumani, Armando Teixeira-Pinto, Clara K. Chow, Jaroslaw Harezlak, Emmanuel Stamatakis

**Affiliations:** 1 Mackenzie Wearables Research Hub, Charles Perkins Centre, The University of Sydney, Sydney, NSW Australia; 2 School of Health Sciences, Faculty of Medicine and Health, The University of Sydney, Sydney, New South Wales Australia; 3 Charles Perkins Centre, The University of Sydney, Sydney, New South Wales Australia; 4 Department of Sports Science and Clinical Biomechanics, Danish Centre for Motivation and Behaviour Science (DRIVEN), University of Southern Denmark, Odense, Denmark; 5 School of Public Health, Faculty of Medicine and Health, University of Sydney, Sydney, New South Wales Australia; 6 Department of Cardiology, Westmead Applied Research Centre, Westmead Hospital, University of Sydney, Sydney, New South Wales Australia; 7 Department of Epidemiology and Biostatistics, School of Public Health, Indiana University, Bloomington, IN United States of America

**Keywords:** Lifestyle physical activity, All-cause mortality, Machine learning

## Abstract

**Background:**

Vigorous intermittent lifestyle physical activity (VILPA) completed through normal daily living may offer a time-efficient avenue to accrue physical activity in a behaviourally sustainable manner. However, no research to date has explored its association with mortality in a nationally representative population. This study aimed to examine the dose-response association between VILPA and mortality risk in a nationally representative sample of US adults.

**Methods:**

This study included a nationally representative sample of 3293 US adults from the 2011-14 National Health and Nutrition Examination Survey (NHANES) who self-reported no participation in structured exercise (52.3% female; mean age: 50.7 [SD: 16.6 years]. The dose-response relationship between VILPA and all-cause mortality was estimated using multivariable-adjusted cubic splines. Average daily frequency (bouts/day) and duration (minutes/day) of VILPA bouts lasting up to one minute were measured using a wrist-worn accelerometer.

**Results:**

Over the mean (SD) 6.7 (1.4) year follow-up period, 290 all-cause mortality events occurred. Compared to the referent point (0 bouts per day), there was an L-shaped dose-response association where the median frequency (5.3 bouts per day) was associated with a 44% lower risk of all-cause mortality (HR: 0.56; 95% CI: 0.39, 0.82). The dose response curve was less steep beyond approximately 8 bouts per day (HR: 0.46; 95% CI: 0.28, 0.77). Findings for the median frequency of VILPA bouts (5.3 bouts per day) remained consistent after excluding participants with poor health (HR: 0.51: 95% CI: 0.29, 0.87) and those who completed no VILPA (HR: 0.57: 95% CI: 0.38, 0.86). When excluding adults with prevalent cardiovascular disease or cancer at baseline (*n* = 2,731, 152 events), the dose-response relationship was similar, although the 95% CIs crossed unity for most of the curve (e.g. median frequency of VILPA bouts HR: 0.68: 95% CI: 0.41, 1.11).

**Conclusions:**

In a nationally representative sample of US adults, short bursts of intermittent vigorous physical activity were associated with a lower risk of mortality. Excluding those with prevalent cardiovascular disease and cancer led to attenuated dose-response curves and wider confidence intervals, suggesting that the observed relationships may, at least partly, be driven by existing disease-induced reverse causation. While these results highlight some potential of VILPA as a time-efficient source of activity, additional observational studies with longer follow up and larger sample sizes are warranted.

**Supplementary Information:**

The online version contains supplementary material available at 10.1186/s12966-026-01876-2.

## Background

Physical inactivity is a major risk factor for all-cause, cardiovascular, and cancer mortality [1], presenting an unresolved public health challenge. Despite decades of public health efforts, the prevalence of insufficient physical activity has grown by 19% from 2010 to 2022, impacting 31% of adults globally [2]. Both previous [3, 4] and current iterations [5, 6] of physical activity guidelines are largely derived from self-report questionnaires, primarily capturing activities lasting > 10 min. Recent guidelines such as the 2018 Physical Activity Guidelines for Americans [5] and the World Health Organization 2020 Guidelines on Physical Activity and Sedentary Behavior [6], promote “all activity counts” messaging, implicitly recommending short bouts of physical activity. These recommendations were primarily informed by cross-sectional studies that generically assessed non-contextual and non-intensity-specific bouts lasting < 10 minutes [7], with no direct evidence of the role of physical activity coming from very short bouts.

Recent wearable based evidence from a sample of non-exercising adults in the UK Biobank has highlighted the potential health value of such brief bursts, specifically in the form of vigorous intermittent lifestyle physical activity (VILPA: bouts lasting up to 1 min each) and moderate-to-vigorous intermittent lifestyle physical activity (MV-ILPA: bouts < 3 min) [8–11]. For example, 4.4 min per day of VILPA was associated with a 26–30% lower risk of all-cause and cancer mortality and a 32–34% lower risk of cardiovascular mortality [8]. Such studies incorporating wearable devices and novel machine learning methods [8–12] permit the study of high-resolution “micro-patterns” of physical activity [8, 9], such as VILPA and MV-ILPA outlined above. This more granular examination of physical activity patterns was not previously possible in studies relying on self-report-based measures or even previous generation accelerometry studies [13] with lower data resolution (1 min epoch).

From a behavioural standpoint, intermittent short bursts of incidental physical activity offer a theoretical advantage as they can be easily integrated into day-to-day life by individuals who face common barriers to traditional exercise such as a lack of time, motivation, or access to facilities [14]. This has major public health relevance considering only 20–25% of adults engage regularly in leisure time physical activity [15, 16]. Previous studies [8, 10–12] exploring the relationship between VILPA and mortality used the UK Biobank, a cohort that is more educated, has fewer chronic health conditions, less obesity, and is more socioeconomically advantaged compared to the general population [17]. To date, no study has examined the association of brief intermittent bursts of vigorous physical activity incorporated into daily living activities with mortality among more diverse adults with lower levels of physical activity.

The aim of this study is to examine the dose-response association between daily VILPA frequency and duration with all-cause mortality risk in a nationally representative sample of US adults. By examining the relationship between VILPA and mortality in adults with a more heterogeneous composition of ethnicity, health status, and lifestyle behaviours, this study improves the external validity of the previous VILPA findings and improves the generalisability of these relationships across more diverse populations.

## Methods

### Study design and participants

This prospective cohort included non-exercising adults aged ≥ 20 from the National Health and Nutrition Examination Survey (NHANES) during 2011–2014. NHANES is an ongoing annually collected survey that employs a multi-stage probability sampling design to capture the health and lifestyle habits representative of the noninstitutionalized US population [18]. This sampling design includes oversampling certain subgroups, such as racial/ethnic minorities, low-income individuals, and older adults, to improve the precision of prevalence estimates in these subgroups when incorporating survey weights that account for oversampling, non-response and poststratification [18]. All NHANES protocols have been approved by the National Center for Health Statistics (NCHS) and informed consent was collected from each participant (NCHS Ethics Review Board protocol number #2011-17).

From 2011 to 2014, during the mobile examination visits, a sub-sample of 14,693 participants from the main NHANES survey were invited to wear wrist-worn trackers (ActiGraph model GT3X+, Pensacola, FL) on their non-dominant hand for 7-days [18–20]. Participants were instructed to wear the devices for 24 h per day while sleeping and awake, including while in the bath, shower, or when swimming. The devices were programmed with a sampling rate of 80 Hz and distributed to participants at the end of the mobile examination visits [18–20]. To be included in our analyses, participants must have worn the device at least three valid monitoring days (> 16 h), including at least one weekend day [8–11, 21, 22]. We excluded all subjects who incorrectly wore the device on the dominant wrist, reported mobility issues such as difficulty walking, or required equipment to walk (Supplementary Fig. 1) [23].

As in previous studies [8–12, 24, 25], to enable the examination of the health effects of incidental physical activity, we included only participants who reported no moderate or vigorous leisure time exercise participation in a typical week, as captured by the Global Physical Activity Questionnaire at baseline [18–20]. The examples provided include participating in recreational sport activities such as running, basketball, golf, bicycling, or walking (Supplementary Table 1).

### Ascertainment of mortality

Mortality data was ascertained via the National Center for Health Statistics where participant information was linked to the National Death Index. The underlying cause of death was reported using the International Statistical Classification of Diseases and Related Health Problems (ICD-10) code. All-cause mortality included deaths from all known causes (Supplementary Table 2). The follow-up period was defined as the duration from the mobile examination (i.e., date of accelerometry collection) to the date of death or December 31st, 2019 for those without a mortality event. Analyses were performed between January 1st and August 1st 2024.

### Device-measured physical activity classification

As described in detail previously [8–12, 26], we used a validated two-stage random forest intensity classification schema to determine specific activities and intensity (84.6% accuracy across all intensities: Supplementary Methods). In brief [8–12, 26], this classifier first categorises each 10-second epoch as one of four activity classes including: sedentary (lying or sitting still), standing utilitarian (for example, ironing a shirt, washing dishes), walking (for example, gardening, active commuting, mopping floors), or running/high energetic activities (for example, active playing with children). At the second stage, these activity classes are then assigned one of four intensities including: sedentary, light (< 3 METs), moderate (≥ 3 to < 6 METs), and vigorous activities (≥ 6 METs). Walking activities were classified using the raw magnitude of wrist movement acceleration using milligravity (mg), defined as light (< 100 mg), moderate (≥ 100 mg) and vigorous (≥ 400 mg) intensity. The vigorous intensity definition and bout length selection of up to 1 min in duration was based on both laboratory data and the naturally occurring bout length in the present and previous studies [8, 10–12]. The total VILPA duration and frequency of bouts was estimated for each participant across the days with valid wear-time. Additional details regarding the VILPA definition and bout length selection are provided in the Supplementary Methods.

### Statistical analysis

The frequency and daily duration for VILPA were truncated at the 2.5 and 97.5 percentile to reduce the potential influence of sparse data [8, 21]. We estimated multivariable adjusted absolute risk for all models using log-link Poisson Regression with robust standard error to calculate the dose-response association of VILPA frequency and daily duration with mortality risk [12, 21]. We assessed the dose-response association between VILPA with mortality risk using restricted cubic splines with 0 bouts per day and 0 min per day for daily duration as the reference (knots placed at the 10th, 50th, and 90th percentile) [8, 9]. Time-to-event associations of VILPA with mortality risk were estimated using Cox proportional hazards models. To provide point estimates for the dose-response associations, we report the hazard ratio (HR) for the median of each exposure and the minimal volume dose (50% of the optimal dose) [8, 21, 27]. We identified the inflection point [28] in the dose-response curve by examining the difference between successive HRs (Supplementary Fig. 2–3). To account for the complex survey design, we applied the weights to all models which ensures the estimates are representative of the noninstitutionalized US population [18]. All models met the Cox proportional hazards assumptions.

As in previous analogous analyses [8, 9, 12, 21], we adjusted for self-reported age, gender, education, race/ethnicity, socioeconomic status (income-to-poverty ratio), fruit and vegetable intake (servings per day), accelerometry estimated sleep duration, medication (glycemic control, lipid-lowering, or blood pressure), discretionary screen time, previous cancer, previous cardiovascular disease, previous diabetes, and familial history of cardiovascular disease or type-two diabetes [8, 9, 12, 21]. A detailed description of each covariate is provided in Supplementary Table 3. The models with VILPA frequency (bouts per day) as the exposure were additionally adjusted for total physical activity energy expenditure (PAEE) [29, 30]. The models with VILPA duration (minutes per day) as the exposure were additionally adjusted for light and moderate PAEE and the daily VILPA duration from bout lengths > 1 minute [8–10, 12]. All missing covariates were estimated using a random forest multiple imputation by chained equations model (5 imputed datasets).

### Sensitivity analyses

To test for the possibility of sex-based differences, we tested for multiplicative interaction and the relative excess risk due to interaction. We completed several sensitivity analyses to examine the robustness of our findings to reverse causation and other possible biases. First, we excluded underweight participants (BMI of < 18.5), participants reporting a self-perceived rating of poor health or participants with prevalent CVD or cancer. Second, we created additional models using alternative reference points (5th, 25th, and 50th percentile) in the dose-response association [9, 21] or excluding individuals with extreme VILPA values. Third, we repeated the main analyses with adjustment for cardiometabolic risk factors, which are possible mediators in the association between physical activity and mortality risk [9, 31]. We provide a sensitivity analysis adjusting for a more comprehensive diet quality indicator (Healthy Eating Index 2015) [32] to address potential confounding by other dietary factors [23]. In another analysis, we excluded individuals with missing covariate information rather than applying multiple imputations to address potential imputation bias [33]. To test for possible misclassification of non-exercisers, we completed a sensitivity analysis repeating the main analyses with two alternative non-exerciser definitions (a) excluding adults who self-reported only completing recreational VPA and (b) excluding adults who self-reported ≥ 1 day per week of recreational MVPA). We also conducted a sensitivity analysis by treating accidents and residual deaths (i.e., contains a mixture of accidental and other causes of deaths that do not have clear links to physical inactivity) as a competing interest using Fine-Gray sub-distribution hazard models (see Supplementary Table 2 for ICD-10 Codes).

### Additional analyses

To enable a direct comparison of VILPA associations with our previous work in middle-aged adults [8–12], we repeated the core analysis in adults ≥ 40 years old. Additionally, to assess the possibility of unmeasured confounding, we calculated e-values for each model [34]. There was no problematic multicollinearity present in any of the models (Supplementary Table 4).

All analyses and graphics in this study were conducted using the survival, survey, RMS, mice, and ggplot2 packages in R statistical software (v.4.3.1). This study followed the Strengthening the Reporting of Observational Studies in Epidemiology Guidelines (STROBE).

## Results

### Participant characteristics

The final analytical sample included 3293 non-exercising adults (52.3% female) with a median age of 50.7 [SD: 16.6] years (Table 1). The sample included a substantial proportion of adults (44%) consuming medication for glycemic control, lipid lowering, or blood pressure management, with 9.8% reporting a history of cardiovascular disease and 9.3% reporting a history of cancer. The majority of participants had complete covariate information (92.9%). A large proportion all vigorous bouts lasted < 1 min in duration (95% of all bouts) with a median bout length of 10 s [IQR: 20 s]. The median VILPA frequency and duration were 5.3 [IQR: 8.9] bouts per day and 1.1 min [IQR: 2.09] per day, respectively. Participants had a median wear time of 23.1 [IQR: 1.2] hours per day. Over a 6.7-year median follow-up period, there were 290 all-cause mortality events, corresponding to 22,183 person-years.

**Table 1:** Participant baseline characteristics by VILPA duration (min/day)

VILPA duration quartiles (min/day)	0	< 0.4	0.4–1.1	1.1–2.5	> 2.5	Overall
Sample size (*n*)	129	791	791	791	791	3,293
All-cause mortality, *n* (%)	59 (45.7)	129 (16.3)	60 (7.6)	26 (3.3)	16 (2.0)	290 (8.8)
Follow up, years	5.8 (1.8)	6.5 (1.6)	6.8 (1.4)	6.8 (1.3)	6.9 (1.2)	6.7 (1.4)
Age, years	72.6 (9.2)	62.4 (14.3)	49.9 (14.9)	45.2 (14.4)	41.8 (13.2)	50.7 (16.6)
Sex, Female *n* (%)	68 (52.7)	468 (59.2)	455 (57.5)	404 (51.1)	327 (41.3)	1,722 (52.3)
Smoking, *n* (%)						
Current	24 (18.6)	167 (21.1)	235 (29.7)	187 (23.7)	190 (24.1)	803 (24.4)
Never	61 (47.3)	397 (50.3)	399 (50.4)	451 (57.2)	451 (57.1)	1,759 (53.5)
Previous	44 (34.1)	226 (28.6)	157 (19.8)	151 (19.1)	149 (18.9)	727 (22.1)
Alcohol, *n* (%)^1^	53 (41.1)	279 (35.3)	223 (28.2)	230 (29.1)	203 (25.7)	988 (30.0)
Education, *n* (%)						
Less than 9th grade	21 (16.3)	105 (13.3)	64 (8.1)	72 (9.1)	116 (14.7)	378 (11.5)
9-11th grade	30 (23.3)	135 (17.1)	119 (15.0)	138 (17.4)	154 (19.5)	576 (17.5)
High school graduate/GED or equivalent	28 (21.7)	179 (22.6)	194 (24.5)	177 (22.4)	194 (24.5)	772 (23.4)
Some college or AA degree	34 (26.4)	232 (29.3)	267 (33.8)	242 (30.6)	217 (27.4)	992 (30.1)
College graduate or above	16 (12.4)	140 (17.7)	147 (18.6)	162 (20.5)	110 (13.9)	575 (17.5)
Ethnicity, *n* (%)						
Mexican American	5 (3.9)	66 (8.3)	96 (12.1)	119 (15.0)	172 (21.7)	458 (13.9)
Hispanic	10 (7.8)	71 (9.0)	91 (11.5)	98 (12.4)	101 (12.8)	371 (11.3)
White	69 (53.5)	392 (49.6)	322 (40.7)	272 (34.4)	228 (28.8)	1,283 (39.0)
Black	39 (30.2)	203 (25.7)	185 (23.4)	167 (21.1)	168 (21.2)	762 (23.1)
Asian	5 (3.9)	50 (6.3)	74 (9.4)	108 (13.7)	108 (13.7)	345 (10.5)
Multi-Racial	1 (0.8)	9 (1.1)	23 (2.9)	27 (3.4)	14 (1.8)	74 (2.2)
Diet, serving per day of fruits and vegetables	4.3 (2.8)	4.2 (2.9)	4.3 (3.1)	4.3 (2.8)	4.4 (3.6)	4.3 (3.1)
Income to poverty ratio	2.2 (1.4)	2.4 (1.6)	2.2 (1.6)	2.3 (1.6)	2.1 (1.5)	2.2 (1.6)
Consuming medication for cholesterol, glycemic control and blood pressure, *n* (%)	113 (87.6)	529 (66.9)	358 (45.3)	268 (33.9)	181 (22.9)	1,449 (44.0)
No previous cardiovascular disease, *n* (%)	72 (55.8)	645 (81.5)	741 (93.7)	750 (94.8)	763 (96.5)	2,971 (90.2)
No previous cancer, *n* (%)	91 (70.5)	668 (84.5)	726 (91.8)	743 (93.9)	760 (96.1)	2,988 (90.7)
No familial cardiovascular disease, *n* (%)	111 (86.0)	694 (87.7)	686 (86.7)	719 (90.9)	719 (90.9)	2,929 (88.9)
No familial diabetes, *n* (%)	80 (62.0)	431 (54.5)	433 (54.7)	504 (63.7)	483 (61.1)	1,931 (58.6)
LPA, min/day	46.0 (17.5)	59.5 (21.0)	66.6 (21.1)	71.5 (20.9)	74.6 (21.2)	67.2 (22.0)
MPA, min/day	1.8 (3.9)	6.0 (7.5)	13.1 (10.7)	19.0 (12.6)	29.4 (16.7)	16.3 (15.0)
Sleep, min/day	359.2 (134.9)	365.0 (127.1)	386.2 (113.8)	388.1 (110.9)	391.0 (104.9)	381.7 (115.8)
Body mass index, kg/m^2^	29.4 (6.6)	30.2 (6.9)	30.0 (6.9)	29.1 (6.5)	28.1 (5.9)	29.4 (6.6)

*VILPA* Vigorous intermittent lifestyle physical activity, *MPA* Moderate intensity physical activity, *LPA* light intensity physical activity

The columns breakdown corresponds to the total volume of VILPA. Values represent mean (standard deviation) unless specified otherwise

^1^Alcohol consumption measured 12 drinks/year (1 drink = 12 oz. beer/ 5 oz. glass of wine/ one and a half oz. of liquor)

### VILPA and all-cause mortality risk

In the fully adjusted analytical model, both VILPA frequency and duration displayed an L-shaped association with lower all-cause mortality risk. The minimum daily VILPA frequency (0 bouts per day, 9.6% of the sample) and median (5.3 bouts per day) frequency of VILPA were associated with an absolute risk of 95.7 (95% CI: 55.3, 165.4) and 55.0 (95% CI: 33.5, 90.2) per 10,000 person-years; respectively (Supplementary Fig. 4A). The minimum daily VILPA duration (0 min per day; 25.1% of the sample) was associated with an absolute risk of 89.8 (95% CI: 52.5, 153.4) per 10,000 person-years. The median daily VILPA duration (1.1 min per day) had an absolute risk of 55.7 (95% CI: 34.0, 91.3; Supplementary Fig. 4B) per 10,000 person-years.

**Fig. 1:**
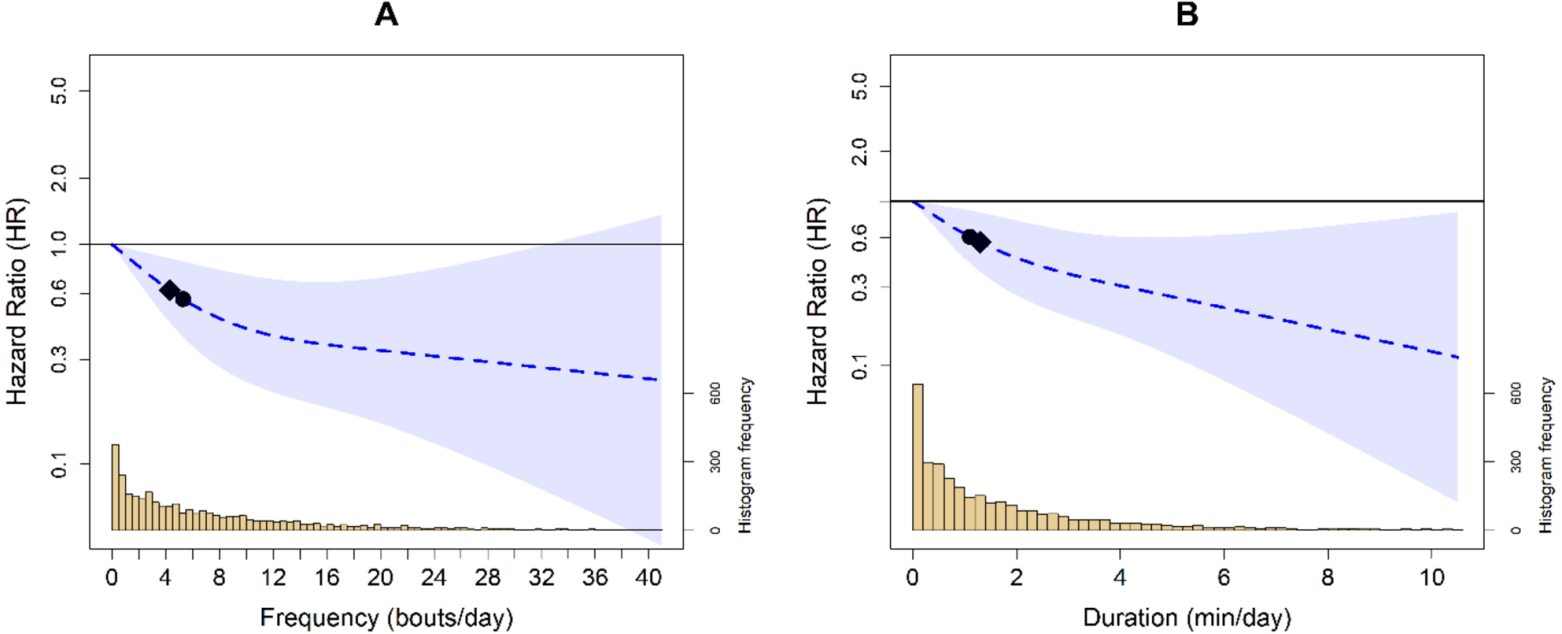
Survey adjusted dose response curves of daily VILPA frequency (**A**) and duration (**B**) with all-cause mortality (*n* = 3293; events = 290). Legend: Analyses were adjusted for sex, age, income, education, ethnicity, fruit and vegetable consumption, smoking history, physical activity energy expenditure from LPA and MPA, alcohol consumption, sleep duration, discretionary screentime, medication use (glycemic control, blood pressure, cholesterol), family history of diabetes and CVD, and previous history of CVD, diabetes, cancer. VILPA bout (**A**) was further adjusted by energy expenditure by vigorous intensity and VILPA duration (**B**) was further adjusted for VILPA bouts over 1-min. All analyses excluded participants who had an event in the first year of follow-up. All analyses were adjusted for strata, cluster, and survey weights. Reference was set to zero for VILPA frequency and duration. Diamond, minimal dose, as indicated by the ED50 statistic which estimates the daily duration of VILPA associated with 50% of optimal risk reduction. Circle, HR associated with the median VILPA value

Compared to the VILPA frequency referent data point (0 bouts per day), we observed an L-shaped dose-response association between VILPA frequency and all-cause mortality risk (Fig. 1A). The median daily VILPA frequency (5.3 bouts per day) was associated with an HR of 0.56 (95% CI: 0.39, 0.82) for all-cause mortality. The slope of the dose-response curve for VILPA bouts per day began to plateau beyond approximately 8 bouts per day, corresponding to a 54% lower risk of all-cause mortality (HR: 0.46; 95% CI: 0.28, 0.77; Supplementary Fig. 2). Compared to the referent minimum daily VILPA duration (0 min per day), there was a near-linear association with all-cause mortality risk (Fig. 1B). The median daily duration of VILPA (1.1 min/day) was associated with an HR of 0.61 (95% CI: 0.42, 0.88). The slope of the dose-response relationship began to plateau beyond approximately 2 min per day, corresponding to an HR of 0.55 (95%CI: 0.27, 0.76; Supplementary Fig. 3). The minimal dose for VILPA bouts (4.3 bouts per day) and daily duration (1.3 min per day) corresponded to an HR of 0.62 (95%CI: 0.44, 0.86) and 0.57 (95%CI: 0.37, 0.85), respectively.

**Fig. 2:**
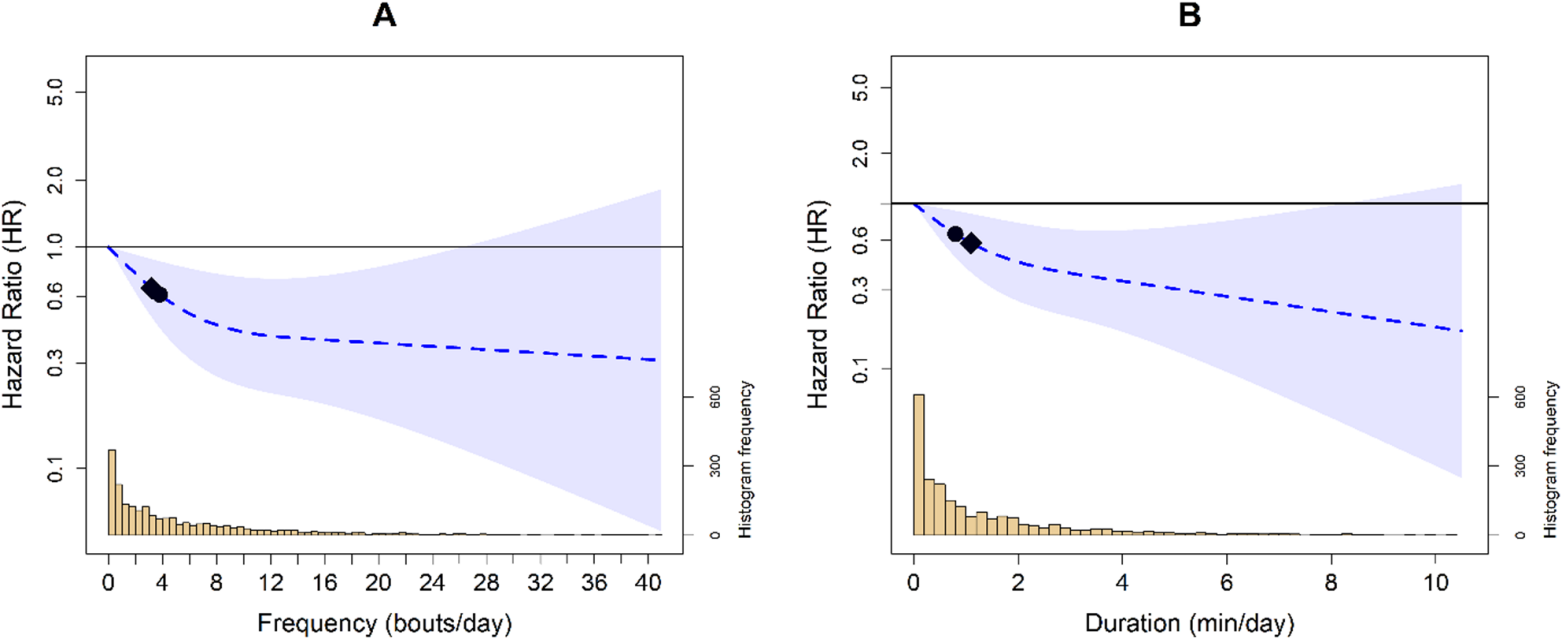
Survey adjusted dose response curves of daily VILPA frequency (**A**) and duration (**B**) with all-cause mortality (n = 2276; events = 272) in adults ≥40. Legend: Analyses were adjusted for sex, age, income, education, ethnicity, fruit and vegetable consumption, smoking history, physical activity energy expenditure from LPA and MPA, alcohol consumption, sleep duration, discretionary screentime, medication use (glycemic control, blood pressure, cholesterol), family history of diabetes and CVD, and previous history of CVD, diabetes, and cancer. VILPA bout (**A**) was further adjusted by energy expenditure by vigorous intensity and VILPA duration (**B**) was further adjusted for VILPA bouts over 1-min. Reference was set to zero for VILPA frequency and duration. All analyses excluded participants who had an even in the first year of follow-up. All analyses were adjusted for strata, cluster, and survey weights. Diamond, minimal dose, as indicated by the ED50 statistic which estimates the daily duration of VILPA associated with 50% of optimal risk reduction. Circle, HR associated with the median VILPA value

In adults ≥ 40 years old, the association between VILPA frequency and duration was attenuated, displaying a clearer L-shape relationship with all-cause mortality (Fig. 2). Compared to the minimum VILPA frequency (0 bouts per day) and duration (0 min per day), the median VILPA frequency (3.8 bouts per day) and duration (0.8 min per day) were associated with an HR of 0.61 (95%CI: 0.43, 0.86) and 0.65 (95%CI: 0.47, 0.90), respectively. The minimal dose for VILPA frequency (3.2 bouts per day) and daily duration (1.1 min per day) was associated with an HR of 0.65 (95%CI: 0.48, 0.88) and 0.58 (95%CI: 0.39, 0.87), respectively.

### Sensitivity analyses

The associations between VILPA and all-cause mortality did not significantly differ when excluding individuals with poor self-rated health (Supplementary Fig. 5). When excluding adults with prevalent disease (Supplementary Fig. 6), the dose-response curves were similar to main analyses but 95%CIs crossed unity for most of the curve. The associations were not materially affected when adjusting for cardiometabolic health (Supplementary Figs. 7–8), diet quality (Supplementary Fig. 9), or when excluding individuals with upper and lower ranges of VILPA (Supplementary Figs. 10–12). The associations were unchanged when treating accidents as a competing interest, while treating residual deaths as a competing interest largely attenuated the associations (Supplementary Figs. 13–14). There were no materially different results when using alternative referent points (Supplementary Fig. 15–17), different definitions of non-exercisers (Supplementary Figs. 18–19), or when excluding individuals with missing covariate information (Supplementary Fig. 20).

### Additional analyses

There was no significant interactive or synergistic association between sex and VILPA with all-cause mortality (Supplementary Table 5). E-values indicated that to attenuate the primary findings to null, the association of an unmeasured confounder for VILPA frequency and duration would need to be 3.59 (lower 95% CI: 1.96) and 3.59 (lower 95% CI: 2.00), respectively (Supplementary Table 6).

## Discussion

This is the first US-based study examining the associations of brief intermittent vigorous activity embedded into daily living with mortality risk. Compared to individuals who accumulated no daily VILPA bouts, accumulating 5–6 very brief bouts lasting < 1 min each, was associated with a 42–47% lower risk of all-cause mortality. We also identified a near-linear inverse association between VILPA duration and all-cause mortality risk, whereby relatively modest amounts of VILPA such as the median duration of just over one minute, was associated with a 39% lower risk of all-cause mortality compared with no daily VILPA. However, when participants with prevalent CVD and cancer were excluded, the dose-response curves were less steep and the 95% confidence intervals were widened and crossed unity, a finding that raises the possibility that existing disease-induced reverse causation may partly explain some of the observed associations. We also note that these results should not be interpreted as evidence that small amounts of VILPA may act as a substitute for established physical activity guidelines.

Previous physical activity research using the US representative NHANES data has primarily focused on context-agnostic total physical activity in the form of total daily steps [35, 36] or MVPA [13, 37–41] and limited exploration of bouts lasting < 10 min in duration [13, 42]. To date, only two studies have examined the association between short bouts of MVPA (i.e., 5–10 min in duration) with all-cause mortality using prospective data [13, 42]. This relatively underexplored aspect of physical activity is important, as the vast majority of adults complete their daily moderate or vigorous physical activity in brief bouts lasting < 3 min in duration (e.g. 88% of all MVPA bouts in the UK Biobank [9, 43], and 92% of bouts in MVPA NHANES). This US study is the first, to our knowledge, to examine the dose-response associations of brief bouts of VILPA [8] lasting < 1 min in duration with all-cause mortality in a population representative sample.

Similar to the previous work in the UK Biobank [8, 9, 12], we demonstrate a beneficial L-shaped dose-response association of VILPA frequency and duration with mortality risk. In comparison to the UK Biobank, our findings indicate a larger magnitude of risk reduction per exposure unit. For example, compared to those completing no daily VILPA, the median VILPA duration of 4.4 min per day in the UK Biobank was associated with a 38% lower risk of all-cause mortality [8], an effect size that was elicited in NHANES from 1.1 min per day. Moreover, VILPA frequency demonstrated steep beneficial L-shaped association with all-cause mortality where the steepness of the dose-response curve began to diminish beyond 8 bouts per day, associated with a 54% lower risk of all-cause mortality. Of note, the VILPA bouts in NHANES occurred less frequently (Median: 5.3 [IQR: 8.9] bouts per day) and were shorter (Median: 10 s [IQR: 20 s] each) when compared to the UK Biobank (Median [IQR] 10.1 [12.8] bouts per day and lasting 25 [6] seconds each).

The noticeable difference between the observed effect size for VILPA in the NHANES dataset and the UK Biobank may be partially attributed to variations in sociodemographic and lifestyle characteristics between the cohorts and the different prevalence of vigorous physical activity [17, 44]. For example, compared to the non-exercisers in UK Biobank participants, the current NHANES sample had a higher average BMI (29.4 vs. 27.6), contained over twice the proportion of current smokers (24.4% vs. 9.2%), and completed approximately half the total daily MVPA (18.4 min vs. 30.3 min/day) [8, 9, 12]. The NHANES cohort also had nearly twice the prevalence of pre-existing CVD (9.8% vs. 5.3%) and a slightly higher prevalence of cancer (9.3% vs. 9.0%) compared to the UK Biobank [8]. These differences in cohort profiles likely reflect a combination of the high prevalence of unhealthy behaviours in the US [44] and the non-representative nature of the UK Biobank, which compared to the general UK population, is more socioeconomically advantaged, less obese, less likely to smoke or drink, and reported fewer self-reported health conditions [8, 9, 12, 17]. These collective differences may contribute to a lower fitness and functional capacity in the NHANES sample, potentially resulting in a greater relative benefit from vigorous physical activity. Additional evidence from the UK Biobank further supports this in the context of incidental physical activity, wherein inactive adults achieved up to two-fold lower mortality risk from intermittent physical activity when compared to adults meeting the physical activity guidelines [43].

This study expands on previous findings [13, 39, 40] among US adults, by demonstrating meaningfully lower risk associated with modest amounts of vigorous-intensity physical activity accrued through very short bouts. Previous research has shown greater cardiorespiratory benefits from brief bursts of high-intensity training (HIIT) in obese or inactive adults when compared to healthy weight active adults [45]. These benefits include various cardiometabolic risk factors that additively contribute to mortality risk, including improved cardiorespiratory fitness, endothelial function, body composition, blood pressure, and glycemic control [45, 46]. While no current recommendations exist for the frequency of vigorous bouts per day, our findings align well with previous evidence suggesting that as little as 3–6 bouts per day of high-intensity movement lasting for 6–20 s (e.g., stair climbing) can improve cardiometabolic profiles in both young and older adults [47–49]. While we observed a plateau for VILPA duration (2 min/day) and frequency (8 bouts/day), these findings should be interpreted cautiously as they may represent an artifact of data sparsity rather than a true biological ceiling. The magnitude of risk reduction observed in our study is also substantial and the biological feasibility of these results warrants careful consideration. Given the relatively less healthy sample, and the attenuation of the associations after excluding those with CVD and cancer, it remains possible that underlying health differences may have, at least partly, contributed to the observed dose-response associations.

The present results highlight some promise for brief, intermittent activity especially considering that the majority of US adults may not be keen, able, or willing to engage in regular structured exercise [50]. While these behaviours do not replace or equate to established physical activity guidelines, they may offer an accessible opportunity to increase movement across broad segments of the population. Previous qualitative work [14], indicates that interventions integrating such brief activity bursts into everyday tasks (e.g., active commuting, household chores, or office work) without the need for dedicated time or equipment may have feasibility advantages over traditional exercise. Commonly reported VILPA activities in a free-living environment include very fast walking, incline walking, short running bursts, outdoor gardening, carrying heavy loads during household chores, or energetic playing with children [9]. If supported by additional research, identifying additional VILPA activities that are both widely accessible and practical could create opportunities to inform public health initiatives or interventions aimed at promoting physical activity in those unable or unwilling to complete traditional structured exercise.

### Strengths and limitations

A key strength of this study is the incorporation of survey design weights into our analyses enabling population-representative estimates for non-institutionalized adults in the US. We also employ a novel machine-learning approach that classifies physical activity intensity in 10-second windows [26, 51]. This is distinct from previous NHANES context-agnostic work which has predominantly examined accelerometry-based total amounts of PA accrued through 1–2 min epochs which are unable to capture very short bouts of VILPA [13, 35–42], or self-reported leisure time physical activity [52–55]. We conducted an extensive range of additional analyses to test the robustness of our results to the risk of reverse causation, confounding, and other biases, including the exclusion of prevalent diseases, frail or poor health individuals, and adjustments for cardiometabolic health markers [8, 9, 21, 23, 56]. We also calculated e-values to assess the possibility of unmeasured confounding [34] indicating that for an unmeasured confounder to attenuate the primary findings to null, the association of an unmeasured confounder for VILPA frequency and duration would need to be 3.59 (lower 95% CI: 1.96) and 3.59 (lower 95% CI: 2.00). These results suggest that such a confounder would need to have a strong and unlikely association with both the exposure and outcome to fully account for the observed relationships.

Despite the measures undertaken, the possibility of reverse causation or unmeasured confounding remains. In particular, we demonstrate that when excluding adults with prevalent CVD and cancer at baseline the dose-response relationship was attenuated, likely due to compromised statistical power and the loss of 138 events (48% of all events), given the high prevalence of CVD and cancer in the cohort and modest follow-up period (6.7 years). A similar pattern of attenuation was observed when excluding participants with minimal amounts of VILPA (i.e., < 0.5 m min/day) or using the alternative percentile-defined referent points (e.g., 5th, 25th, 50th), which also suggests the possible influence of reverse causation. Across these analyses, the attenuation was relatively modest, and reverse causation is unlikely to be the primary explanation of the observed dose-response relationships. It is also plausible that other factors such as misreporting, temporal variation of lifestyle factors (e.g., alcohol intake, smoking, obesity), or the high prevalence of chronic diseases at baseline could have influenced the study findings. In the complete-case analysis the overall association for both VILPA duration and frequency cross-unity, indicating that imputation bias may have influenced the underlying findings in our analysis. We acknowledge that the non-exerciser definition is based on a self-report questionnaire [19, 20] that enquires about activities that lasted for at least continuous 10 min, which may theoretically misclassify those participants who engage in recreational activities that are very brief in nature. Another limitation of this study also includes the possibility of incomplete capturing of VILPA using wrist-worn wearables, e.g. the intensity of activities such as walking uphill or carrying objects (e.g., backpacks) may not be fully captured [26], likely leading to an underestimation of true daily VILPA. Such underestimation could in turn bias estimates, leading to an overestimation of the effect sizes per VILPA time unit. The 7-day measurement of physical activity may not reflect true long-term behavioural patterns and may subject the results to higher risk of regression dilution bias due to intra-individual variability.

## Conclusion

VILPA frequency and duration were associated with lower all-cause mortality risk in an L-shaped and linear manner, respectively. These findings should be interpreted cautiously as the dose-response curves crossed unity after excluding those with prevalent CVD and cancer, suggesting underlying health status may, at least partly, explain the primary findings. While these findings highlight some potential of VILPA as a time-efficient and behaviourally sustainable form of activity, further replication in larger cohorts with longer follow-up time is needed. Importantly, these findings should be viewed as a complementary, rather than substitutive, form of physical activity alongside established guidelines.

## Supplementary Information

Supplementary Material 1.

## Data Availability

The data that support the findings of this study are publicly available at [https://wwwn.cdc.gov/nchs/nhanes/].

## Abbreviations

BMI: body mass index
CI: confidence interval
CVD: cardiovascular disease
HEI: Healthy Eating Index
HIIT: high intensity interval training
HR: hazard ratio
ICD-10: International Statistical Classification of Diseases and Related Health Problems, 10th Revision
IQR: interquartile range
METs: metabolic equivalents
mg: milligravity
MV-ILPA: moderate to vigorous intermittent lifestyle physical activity
MVPA: moderate to vigorous physical activity
NCHS: National Center for Health Statistics
NDI: National Death Index
NHANES: National Health and Nutrition Examination Survey
PA: physical activity
PAEE: physical activity energy expenditure
SD: standard deviation
STROBE: Strengthening the Reporting of Observational Studies in Epidemiology
UK: United Kingdom
US: United States
VILPA: vigorous intermittent lifestyle physical activity
VPA: vigorous physical activity
